# Spinal muscular atrophy in the era of newborn screening: how the classification could change

**DOI:** 10.3389/fneur.2025.1542396

**Published:** 2025-05-07

**Authors:** Antonio Varone, Gabriella Esposito, Ilaria Bitetti

**Affiliations:** ^1^Pediatric Neurology, Santobono-Pausilipon Children's Hospital, Naples, Italy; ^2^Department of Molecular Medicine and Medical Biotechnologies, School of Medicine and Surgery, University of Naples Federico II, Naples, Italy; ^3^CEINGE Advanced Biotechnologies “Franco Salvatore”, Naples, Italy

**Keywords:** spinal muscular atrophy, neuromuscular disorders, gene replacement therapy, motor function, newborn screening

## Abstract

Spinal muscular atrophy (SMA) is an autosomal recessive neuromuscular disease caused by deletions or mutations in the *Survival Motor Neuron 1* gene, associated with high morbidity and mortality related to muscle weakness. In recent years, the availability of new disease-modifying therapies and the extension of newborn screening has brought radical changes in the natural history of SMA at all ages. Historically, the classification of SMA has been based on age of onset and achievement of maximum motor milestone. In this new era, the historical classification of SMA by typology is no longer adequate to define the prognosis and type of SMA, nor to guide clinical management and treatment choice. The aim of this work is to discuss the current status of SMA neonatal screening and access to therapies across Europe and propose a new updated nomenclature, more suitable to guide clinicians in the management of SMA patients in the era of newborn screening. In this perspective, we evaluate and analyze the genetic basis of the disease, the current therapeutic landscape, the possible genotypic/phenotypic scenarios and the related clinical management.

## 1 Introduction

Spinal muscular atrophy (SMA) is an autosomal recessive neuromuscular disorder caused by deletions or mutations in the *Survival Motor Neuron 1* (*SMN1*; NM_000344.3) gene. SMA is characterized by loss of the spinal cord alpha motor neurons, causing progressive muscle weakness and skeletal muscle atrophy. The *SMN2* gene, a paralog of *SMN1*, produces low levels of functional SMN protein. Consequently, copy number of *SMN2* is inversely related to the disease severity (1). The incidence of SMA is approximately 1 in 3900-16000 live births, in Europe (2); the estimated prevalence for cases of SMA followed in 35 Italian Reference centers was 2.12/100,000 inhabitants, at the end of 2022 (3). Until the 80s, SMA was divided into two main groups: acute infantile SMA (SMA type I or Werdnig-Hoffmann disease) and chronic infantile SMA (SMA types II and III, or Werdnig-Hoffmann disease, Kugelberg-Welander disease or chronic generalized SMA). Subsequently, in the 90s, SMA was recognized as a monogenic disease (SMA 5q) and was classified into 5 subtypes defined by age of symptom onset and maximum motor function achieved (4).

Since 2016, 3 disease-modifying therapies (DMTs) have been approved: nusinersen (Spinraza™, Biogen, Cambridge, MA), onasemnogene abeparvovec-xioi (Zolgensma™, Novartis Gene Therapies, Bannockburn, IL) and risdiplam (Evrysdi™, Genentech/Roche, South San Francisco, CA). In addition, many countries have introduced newborn screening (NBS) for SMA (5). Availability of DMTs and NBS has brought radical changes in the natural history of SMA at all ages. Consequently, the historical classification of SMA by type is no longer adequate to identify prognosis and type of SMA, nor to direct clinical management and choice of treatment.

The aim of this work is to propose a new updated classification suitable to guide clinicians in the management of SMA patients in the era of NBS.

## 2 Spinal muscular atrophy (SMA): clinical and genetic overview

### 2.1 Clinical features and historical classification of SMA patients

In 1991, an international consortium on spinal muscular atrophy sponsored by the Muscular Dystrophy Association (MDA) formalized a classification scheme for the different phenotypes of SMA. This classification highlighted three types of SMA based on the highest level of motor function (non-sitting, sitting, walking) and age of onset. Subsequent modifications included a type 0 for patients with prenatal onset and a type 4 for adult-onset cases (4). Thus, the classification considered the following subtypes:

- type 0: prenatal onset and life expectancy of a few days.
- type I: Werdnig-Hoffmann disease; onset before 6 months of age and characterized by failure to reach a sitting position.

There are three subtypes of SMA type 1 currently recognized by an international consortium of neuromuscular experts and these subtypes correspond to the ages of onset of weakness observed in patients, from birth to 6 months of age. By combining age of onset and achievement of head control as differentiating factors, the following nomenclature for SMA type 1 subtypes was therefore proposed (6):

1. SMA 1A: the most severe form, with neonatal onset (within the first 2 weeks of life). Affected infants present global weakness, profound hypotonia, feeding difficulties and respiratory failure, failure to acquire head control.
2. SMA 1B: onset of severe generalized weakness and hypotonia by 3 months of age. These affected infants often present with a bell-shaped chest and a paradoxical breathing pattern.
3. SMA 1C: the onset of signs and symptoms in subtype 1C occurs between 3 and 6 months of age. Infants usually acquire head control, but never a sitting position.

Due to the wide clinical variability, several subclassification systems have been also proposed for SMA type 1, such as that proposed by Dubowitz which recognizes nine subtypes (1.1–1.9) (7).

- type II or Dubowitz disease, an intermediate form with onset before 18 months of age and failure to walk.
- type III or Kugelberg-Welander disease, with onset after 18 months of age, global weakness but ability to stand and walk unaided.
- type IV (the adult form).

### 2.2 Molecular basis of SMA

**Figure 1:**
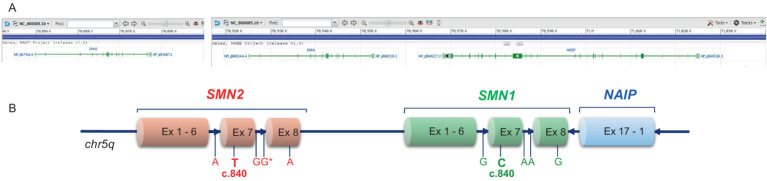
Simplified diagram of the 5q13.2 locus on chromosome 5 containing the SMN1, SMN2 and NAIP genes. **(A)** Screenshot of the chromosomal position of the three genes, according to the chromosome 5 reference GRCh38.p14 primary assembly (NC_000005.10; www.ncbi.nlm.nih.gov/gene; accessed on April 18, 2025). **(B)** Schematic representation of SMN1, SMN2 and NAIP, with the main nucleotide differences between SMN1 and SMN2. In SMN2, the c.840T nucleotide promotes skipping of exon 7 in about 90% of transcripts, thereby significantly reducing the overall SMN protein expression by this gene.

All SMA types are associated with the mutation of the *SMN1* gene that, with its paralog *SMN2* (NM_017411.4) and with the *NLR family apoptosis inhibitory protein/BIRC1* (*NAIP*) gene (NM_004536.2), lies within a segmental duplicated region on chromosome 5q13. Both *SMN1* and *SMN2* encode the ubiquitous protein SMN, a molecular chaperone that provides a platform for RNA and proteins assembly in ribonucleoprotein (RNP) complexes (8). The *NAIP* gene is located about 15 kb downstream to *SMN1*; the two genes are oriented on opposite strands (Figure 1).

A crucial difference between *SMN1* and *SMN2* is the nucleotide c.840 in exon 7, which is a cytosine (C) in *SMN1* and a thymine (T) in *SMN2*. In *SMN2*, c.840 T affects an exonic splicing enhancer and promotes skipping of exon 7 in about 90% of transcripts (SMNΔ7), which generates an unstable/unfunctional SMN protein (9, 10). An additional nucleotide located in exon 8 distinguishes *SMN1* from *SMN2*, being a guanine (G) and an adenine (A), respectively (Figure 1).

Approximately 95% of SMA cases results from biallelic pathogenic sequence variations in the *SMN1* gene, mainly represented by deletions including exon 7 of *SMN1*, or by the point mutation c.840C > T that converts exon 7 of *SMN1* to *SMN2,* or by the presence of a hybrid *SMN1-SMN2* gene. The remaining 5% of SMN-related SMA patients are compound heterozygotes for a *SMN1* deletion and a small nucleotide variant (SNV) on the other *SMN1* allele. Only a single SMA patient with a homozygous mutation in the *SMN1* gene has been reported (11).

In contrast, biallelic loss of *SMN2* is relatively common in the general population (10). However, no SMA cases with biallelic loss of both *SMN1* and *SMN2* have been reported.

Alleles with two or more copies of *SMN1* and *SMN2* also exist in the general population. In SMA patients, *SMN2* copy number influences onset and severity of the disease. Indeed, for patients with ≤ 2 *SMN2* copies, an infantile onset can be predicted; three *SMN2* copies are in many cases associated with phenotypes of intermediate severity; and ≥ 4 copies are mainly predictive of a milder, later onset, SMA phenotype (10).

**Figure 2:**
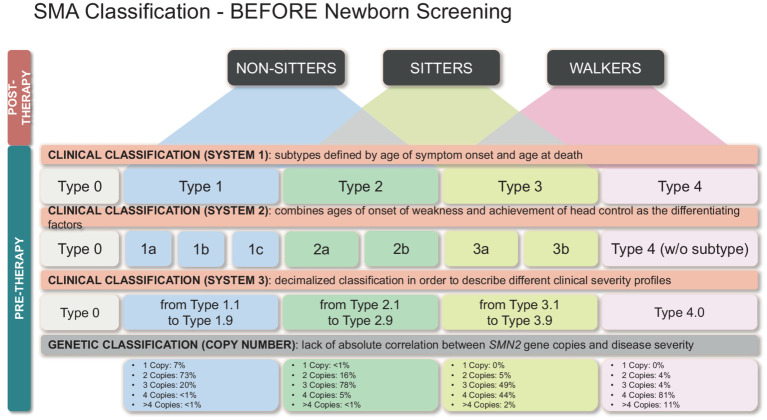
Classification of SMA patients identified by NBS based on genetic and clinical features. Therapeutic options and clinical menagement are also reported.

It should be noted, however, that the number of copies of *SMN2* is not entirely predictive of the subtype, so other factors play a role in determining the age of onset and severity (Figure 2) (12, 13).

Indeed, in SMA patients with two *SMN2* copies, biallelic loss of *NAIP* is a negative disease-severity predictor (14). In contrast, the *SMN2* polymorphism c.859G > C is a positive disease-modifier (15). Also, the *SMN1* c.840C > T pathogenic conversion, in homozygotes and in compound heterozygosity with a deleted *SMN1* allele, is considered predictive of a less severe phenotype also in patients with ≤ 2 *SMN2* copies (10).

### 2.3 Molecular diagnosis of SMA

Genetic testing revealing homozygous *SMN1* deletion/conversion of exon 7 confirms the diagnosis in more than 95% of SMA patients, irrespective of disease severity. Molecular diagnosis is performed through the analysis of genomic DNA that can be extracted from various biological samples, including peripheral blood, saliva, tissues, as well as chorionic villus sampling specimens or amniotic fluid for prenatal test (11, 16). The main molecular methodologies for SMA diagnosis are based on quantitative polymerase chain reaction (qPCR) assays by using probes that specifically target the c.840 nucleotide in exon 7 and discriminate between copy number of *SMN1* and *SMN2*. Widely used is the multiplex ligation-dependent probe amplification (MLPA), which evaluates copy number of exon 7 and 8 of *SMN1* and *SMN2,* and achieves SMA diagnosis and *SMN2* copy number assessment, at the same time; furthermore, it evaluates the number of copies of *NAIP*, which are also related to the disease prognosis, and doses the overall copies of the remaining *SMN1/2* exons thus revealing deletions involving and/or limited to these gene regions. Furthermore, real time qPCR assays performed by using TaqMan hydrolysis probes or LightCycler® probes specifically targeting the *SMN1* exon 7 are exploited (17, 18); similarly, real time qPCR-based assays can also evaluate *SMN2* copy number. Lastly, the AmplideX® SMA Plus Kit (Asuragen, Austin, TX, USA) quantifies copy number of exon 7 of *SMN1* and reports as 0, 1, 2, 3, or ≥ 4 the genomic copies of *SMN2*. This latter kit also identifies the hybrid *SMN1*-*SMN2* gene and detects, in the 3′ untranslated region of *SMN1*, the variants c.*3 + 80 T > G and c.*211_*212del, which are often associated with a *SMN1* duplicated allele, thus allowing identification of the “silent carrier” status (i.e., heterozygous carriers with a deleted *SMN1* allele and a *SMN1* duplication on the other allele); in addition, it identifies the *SMN2* polymorphic variant c.859G > C, which is associated with a milder disease phenotype (19).

These DNA assays have a diagnostic sensitivity and specificity of 95 and 99%, respectively, for SMA (11). In all cases, assay conditions allow determination of *SMN1* and *SMN2* copy number, so that they can also identify heterozygous carrier of *SMN1* deletion. Therefore, diagnostic sensitivity of SMA can increase to >99% by considering that about 5% of SMN-related patients/newborns can be compound heterozygotes for *SMN1* deletion and a small nucleotide variant (SNV) that may cause a frameshift, nonsense, or missense change, on the other *SMN1* allele (11). Consequently, in patients with clinical suspicion of SMA with 1 copy of *SMN1* after a quantitative molecular test, sequencing of the whole coding region of the *SMN1/2* genes is warranted to look for the possible pathogenic SNV. The very rare patients with biallelic SNVs in *SMN1* could remain undiagnosed (20).

Parents of an affected infant are usually heterozygous carriers of *SMN1* deletion/conversion and have a 25% procreative risk of disease recurrence. In these couples, prenatal molecular diagnosis is a realistic option to test the fetus and prevent the birth of an affected child (11, 16). Therefore, prenatal molecular diagnosis is a crucial part of a primary preventive management for families having a child affected by SMA.

## 3 Therapeutic options available for SMA patients

Several highly efficient therapeutic options are currently approved for SMA. The therapies are essentially based on two mechanisms: correction of endogenous *SMN2* splicing to increase the level of the functional SMN protein, and gene replacement therapy.

Two splicing correction therapies are currently available:

Nusinersen (Spinraza™, Biogen/Ionis Pharmaceuticals) is an antisense oligonucleotide that blocks an ISS-N1 splicing silencer in intron 7 of the *SMN2* pre-mRNA, allowing inclusion of exon 7 in the *SMN2* mRNA. The therapeutic protocol includes intrathecal administration of 4 loading doses over 63 days and then a maintenance dose every 4 months, for life. Several studies including all types of SMA patients reported safety, tolerability and clinical efficacy (1). Clinical trials showed that pre-symptomatic infants with three copies of *SMN2* achieved motor milestones similar to healthy infants; infants treated before 6 weeks of age performed better than those treated after 6 weeks of age (21). This evidence indicates that SMA diagnosis by NBS should be made within the first weeks of life.
Risdiplam (Evrysdi, Roche) is a small molecule that crosses the blood–brain barrier and facilitates retention of *SMN2* exon 7 within the mRNA, thereby increasing SMN protein levels. Risdiplam is administered orally daily. Indeed, various clinical studies reported good tolerability and clinical efficacy, and clinical trials demonstrated best results in presymptomatic patients (22).
The SMA gene replacement therapy, onasemnogene abeparvovec-xioi (Zolgensma™; Avexis/Novartis) is a self-complementing adeno-associated virus 9 (AAV9) expressing the *SMN1* cDNA under a cytomegalovirus enhancer/chicken *β*-actin hybrid promoter. The therapy requires a single intravenous injection of 1.1 × 10^14^ viral genomes/kg body weight.

### 3.1 Spinal muscular atrophy therapy in Europe

Until 2020, twenty-nine European countries have access to Nusinersen for 5q SMA through regular reimbursement. In countries such as Denmark, Latvia, Bulgaria, Hungary, Iceland, Ireland, Norway, Finland, reimbursed access has type and/or age restrictions, e.g., <18 years. Some countries have medical/rare disease committees that apply additional clinical inclusion and exclusion criteria. Refund policies in Europen countries are constantly evolving. In Italy, according to the Italian pharmaceutical society (AIFA), Nusinersen is approved for the treatment of all types of 5q SMA patients (23, 24).

Risdiplam has been authorised in the European Union (EU) as Evrysdi since 26 March 2021. The European Commission (EC) extended its approval of Evrysdi to treat SMA infants who are younger than 2 months old, and made the therapy available to treat patients across all age ranges in Europe. Refund policies are not homogeneous throughout Europe. In Italy, according to AIFA, Risdiplam is refundable for the treatment of patients with a clinical diagnosis of SMA Type 1, Type 2 or Type 3 or with one to four *SMN2* copies (25).

According to European Medicines Agency, Zolgensma is indicated for treatment of children with biallelic pathogenic variants in the *SMN1* gene and a clinical diagnosis of SMA Type 1, or asymptomatic children with biallelic *SMN1* mutations and up to 3 copies of the *SMN2* gene. Pre-existing immunity against AAV9, which is the vehicle of the *SMN1* transgene, must be excluded. Reimbursement policy for Zolgensma differs from country to country in Europe. In Italy, according to AIFA, Zolgensma in refundable in patients with a weight range between 2.6 kg and 13.5 kg (26). Treatment indications in France are currently restricted to SMA1 and SMA2 children under 2 years old and weighing less than 12 kg (27). In the UK, onasemnogene abeparvovec has been approved for treatment of patients with genetically confirmed 5q SMA type 1, or for infants up to 12 months old identified pre-symptomatically with up to 3 copies of *SMN2* (28).

## 4 Newborn screening programs for SMA

### 4.1 The current Italian framework

Currently, the Italian health regulatory agency allows treatment with the gene therapy for presymptomatic children with ≤ 3 *SMN2* copies, whereas nusinersen and risdiplam can be administered at all ages to presymptomatic SMA patients with one to four *SMN2* copies. Consequently, in the last years, many Italian regions have implemented NBS programs for early identification of SMA children.

At present, screening is active in 14 Italian regions (29) and covers 33% of newborns

- Abruzzo: screening for SMA started on December 12, 2022.
- Campania: the pilot project that includes the extension to SMA of the list of diseases subjected to screening was approved with Resolution of the Regional Council no. 303 of 06/21/2022 and officially started on April 1, 2023. Screening is carried out in all regional birth points, and is subjected to parental consent.
- Emilia – Romagna: with Resolution No. 1441 of 01/07/2024, the Regional Council of Emilia Romagna established an “Expansion of the neonatal screening panel ex DGR 2260 of 27/12/2018,” which includes not only SMA but, gradually, also aromatic L-amino acid decarboxylase deficiency (AADC deficiency), X-linked adrenoleukodystrophy (X-ALD), severe combined immunodeficiencies (SCID) and X-linked agammaglobulinemia (XLA).
- Friuli – Venezia Giulia: since 2 December 2021, a pilot project for the screening of spinal muscular atrophy has been active, which expired in December 2022 and was subsequently renewed.
- Lazio: following the conclusion and results achieved during the two-year Pilot Project (5 September 2019–5 September 2021) of screening for SMA launched in Lazio and Tuscany, the Lazio Region has ensured continuation of this screening through the regional network of Neonatal Screening services. From 5 September 2021, screening is included in the sampling used for Extended Neonatal Screening (ENS), subject to the collection of informed consent via a specific form.
- Lombardy: at the beginning of July 2023, the Regional Council of Lombardy approved a resolution that provided for the start of screening for SMA starting from September 15, 2023.
- Liguria: launched the Neonatal Screening pilot program for the timely and simultaneous diagnosis of SMA and severe combined immunodeficiencies (SCID) on September 4, 2021.
- Puglia: with Regional Law of April 19, 2021, n. 4, mandatory screening for SMA was introduced in Puglia. Operationally, screening was started on December 6, 2021.
- Piemonte and Valle d’Aosta: On November 14, 2022, a pilot project was launched for the integration of SMA into Extended Neonatal Screening for the early diagnosis of metabolic diseases.
- Tuscany: already in 2018, the Tuscany Region extended neonatal screening to three lysosomal storage diseases and SCID. Subsequently, as well as Lazio, following the conclusion of the experimental project (September 2019 – September 2021), with DGR n. 796 of 2/8/2021, it also included the test for SMA in the screening offered to all newborns in the region.
- Trentino – Alto Adige: from October 1, 2023, SMA has been included among the optional tests of the expanded newborn screening as an additional and free service. On November 1, 2024, the Autonomous Province of Trento also started the same procedure, integrating SMA into the screening panel.
- Veneto: with resolution of the Regional Council n. 1,564 of December 6, 2022, Veneto has provided for the expansion of the panel of pathologies subject to Neonatal Screening also to SMA. From January 1, 2024, the provision is fully operational and involves all regional newborns and all birth centers.
- Sicily: with the approval of the Decree of the Health Department n. 692 of June 6, 2024, the Sicilian Region is also ready to start neonatal screening for spinal muscular atrophy (SMA) on all newborns. This measure allows the Bill 382 of 31 January 2024 “Mandatory neonatal screening for spinal muscular atrophy” to finally become operational.
- Sicily: with the approval of the Decree of the Health Department n. 692 of June 6, 2024, the Sicilian Region is also ready to start neonatal screening for spinal muscular atrophy (SMA) on all newborns. This measure allows the Bill 382 of 31 January 2024 “Mandatory neonatal screening for spinal muscular atrophy” to finally become operational.
- Calabria: with resolution of the Regional Council n. 44 of February 17, 2025, this region has approved SMA NBS program, which is scheduled to start in May-June 2025, in collaboration with the Campania Region.

In three regions, i.e., Basilicata, Marche, and Sardinia, experimental projects are planned to add SMA to the pathologies already diagnosed by the mandatory extended NBS program (29).

However, there are no initiatives currently active or starting for SMA screening in the remaining two Italian regions, namely Molise, and Umbria.

All the Italian regions adopted the same procedure to perform the SMA NBS. Genomic DNA is extracted from dried blood spots (DBS) of the newborns and analyzed to detect, by real time PCR with a probe targeting the c.840C nucleotide in *SMN1*, absence/conversion of the *SMN1* exon 7, which represents the molecular cause of SMA in over 95% of cases. Presumptive positive results are then confirmed by using molecular methodologies that also assess *SMN2* copy number. Indeed, *SMN2* copy number is currently one of the main determinants for therapeutic decision in SMA patients.

Obviously, less than 5% of SMA newborns escapes the screening test, i.e., the compound heterozygotes for the deletion/conversion of exon 7 and another type of *SMN1* mutation (SNV or deletions in other gene regions), or homozygotes for other types of *SMN1* mutations. Currently, the NBS test does not allow identification of heterozygotes for exon 7 deletion/conversion and a putative SNV, which can be intercepted only when the disease symptoms appear, and a specific molecular test is performed (see molecular basis and diagnosis of SMA). Indeed, if or when SMA is clinically determined/suspected and a heterozygous deletion is identified, sequencing of the whole coding region of the *SMN1* gene is warranted to achieve the definitive diagnosis.

### 4.2 NBS for SMA in Europe

As of August 2024, in geographic Europe, 66% of children are screened for SMA at birth. In the EU, 64% of children are screened for SMA at birth.

Currently, a national NBS program is active in Germany, Belgium, Portugal, Sweden, Switzerland, Austria, Luxembourg, Poland, Lithuania, Belgium, Croatia, Denmark, Estonia, Latvia, Netherlands, Norway, Russia, Serbia, Slovakia, Slovenia, Turkey and Ukraine. In France, SMA NBS is approved in Nouvelle Aquitaine, Bordeaux and Grand Est, Strasbourg. In Spain, the NBS includes SMA in Galicia, Balearic and Canary Islands, and pilot studies are ongoing in Madrid and Comunidad Valenciana. In Ireland, SMA has been approved as part of the national NBS program and is awaiting implementation. There are active pilot projects in the Czech Republic, Hungary, Macedonia, Romania, the Thames Valley region (Oxford) in England (30).

## 5 Implications of SMA molecular diagnosis by NBS

In recent years, the new effective drugs available for the treatment of SMA and the introduction of NBS have radically changed the clinical course and prognosis for subject affected by SMA. This requires changes in all aspects of clinical care. Early diagnosis and timely intervention for the disease have led to the emergence of new phenotypes of SMA that deviate from the traditional natural history of the disease, creating a need to study new clinical trajectories to improve the care of affected individuals in the post-treatment era. Hence, traditional classification of the various disease types is currently not applicable. Indeed, with the available therapies, subjects diagnosed as SMA 1 according to the old classification often reach motor milestones, becoming sitters or even walkers. Screened newborns may be asymptomatic, not allowing for adequate phenotype assignment in the traditional classification, based on age of onset. Hence the need to identify new criteria and create a new nomenclature.

### 5.1 Classification of SMA patients identified by NBS

**Figure 3:**
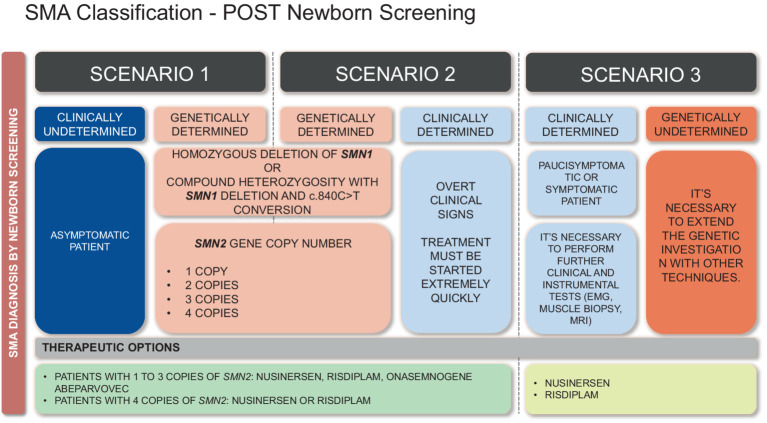
Historical classification of SMA based on age of symptoms onset and highest physical milestone achieved.

In the NBS era, the diagnosis of SMA can be reached even before symptoms appear, in about 95% of cases, while about 5% patients are not detected. Consequently, we must assume that at the end of the screening test three possible situations can arise, which require a novel classification of the infants, as proposed below:

Class 1 infants - Clinically undetermined and genetically determined SMA: asymptomatic infants with homozygous deletion of *SMN1* exon 7 or compound heterozygotes with the c.840C > T conversion, and a variable number of *SMN2* copies (Figure 3).

Class 2 infants - Clinically and genetically determined SMA: symptomatic or paucisymptomatic infants with homozygous deletion of *SMN1* exon 7 or compound heterozygotes with the c.840C > T conversion, and a variable number of *SMN2* copies.

Class 3 infants - Clinically determined but genetically undetermined SMA: newborns/infants with SMA-related symptoms, without a diagnostic genotype for SMA (e.g., no deletion/conversion detected by NBS or heterozygous for *SMN1* deletion/conversion), and a variable number of *SMN2* copies (Figures 2, 3).

### 5.2 Therapies and management of SMA patients identified by NBS

The 3 types of patients classified on the basis of the NBS results should be treated with the available therapeutic options, the choice of which depends on the *SMN2* copy number determination.

Clinically undetermined and genetically determined (Class 1) infants:

- Asymptomatic SMA newborns/infants with 1 to 3 copies of *SMN2*. For these patients, Onasemnogene abeparvovec or Nusinersen or Risdiplam can be prescribed. In children weighing less than 2.6 kg, treatment with Risdiplam or Nusinersen may be initiated with the possibility of subsequent switching to Onasemogene abeparvovec. If pre-existing immunity against AAV9 is found, the choice of initial treatment is between Risdiplam and Nusinersen. It is important to schedule a close follow-up: every 2–3 months until the age of 1 year; every 3–6 months until 2 years; every 6–12 months thereafter. At each check-up, a thorough neurological examination must be performed to identify even the slightest signs of the disease onset. Clinical observation should include the use of standardized rating scales, such as the Children’s Hospital of Philadelphia Infant Test of Neuromuscular Disorders (CHOP INTEND), Hammersmith Neonatal Neurological Examination (HNNE), Hammersmith Functional Motor Scale Expanded for SMA (HFMSE), RULM, Bayley III, Griffiths III. It is possible to start a neuromotor or neuropsychomotor rehabilitation treatment.
- For asymptomatic SMA newborns/infants with 4 copies, it is possible to choose between Risdiplam and Nusinersen. Follow-up should be scheduled every 3–6 months until 1 year of age; every 6–12 months thereafter.
- For asymptomatic SMA newborns/infants with ≥ 5 *SMN2* copies there is no indication for treatment. A follow up may be scheduled.
  In all cases, the decision is made in agreement with the parents after having explained in detail risks and benefits of each therapy; electroneurography may be performed to investigate electrophysiological biomarkers of compound muscle action potential (CMAP).

Clinically and genetically determined (Class 2) infants:

- Symptomatic SMA newborns/infants with 1 copy of *SMN2*: the treatment decision depends on the severity of the condition. If cardiorespiratory dynamics are already too compromised, palliative treatments may be started.
- Symptomatic SMA newborns/infants with 2 or 3 copies of *SMN2*: treatment must be started extremely quickly. One of the three therapies can be chosen. In children weighing less than 2.6 kg, treatment with Risdiplam or Nusinersen may be initiated with the possibility of subsequent switching to Onasemogene abeparvovec. If pre-existing immunity against AAV9 is found, the choice of initial treatment is between Risdiplam and Nusinersen. A motor and respiratory rehabilitation program must be set up immediately. Polysomnography may be useful in assessing the need for non-invasive ventilatory support. It is crucial to schedule a close multidisciplinary follow-up: every 2–3 months until the age of one; every 3–6 months thereafter. Clinical observation should include the use of standardized rating scales (CHOP INTEND, HNNE, HFMSE, RULM, Bayley III, Griffiths III).
- Symptomatic SMA newborns/infants with 4 copies of *SMN2*: considering the phenotypic variability that can be associated with 4 copies of *SMN2*, it is advisable to start treatment with Risdiplam or Nusinersen (31).
- Symptomatic SMA newborns/infants with ≥ 5 SMN2 copies: Nusinersen therapy may be considered.

In all cases, electroneurography may be performed to investigate electrophysiological biomarkers of compound muscle action potential (CMAP).

Clinically determined, but genetically undetermined (Class 3) infants:

- Newborns/infants without homozygous deletion/conversion of *SMN1* exon 7, but with putative SMA-related symptoms. Genetic testing using conventional quantitative methodologies (qPCR, MLPA, etc.) must be requested to evaluate *SMN1* copy number. For infants with a clinical suspicion who resulted heterozygous for *SMN1* deletion, genetic investigation must be extended with other techniques, such as specific amplification of the whole coding region of the *SMN1/2* genes, and subsequent analysis by using conventional Sanger or long read sequencing-based methodologies (32, 33). However, waiting times, in this case, could be long. Therefore, it is necessary to perform further clinical and instrumental tests, such as electromyography, muscle biopsy, magnetic resonance imaging. If all the tests are compatible with the diagnosis of SMA, it would be warranted to undertake off-label therapy with Nusinersen or Risdiplam while waiting for genetic confirmation.

### 5.3 Santobono hospital experience

In our experience, from April 1 2023 to December 31, 2025, 10 positive children (7 females and 3 males) have been identified through NBS in our region. In all cases, the diagnosis of SMA was confirmed by MLPA. However, only 30% of our positive newborns were born with symptoms related to the disease; one of them was admitted to the neonatal intensive care unit. Consequently, the current classification did not allow us to place most of our patients in a specific category of the disease. In fact, asymptomatic newborns could develop symptoms within 6 months of life or later regardless of the number of *SMN2* copies.

According to our proposal, symptomatic or paucisymptomatic newborns should be classified as Class 2 patients, whereas the remaining 60% should be considered Class 1, because they were asymptomatic at birth.

In our settings, first clinical evaluation of positive newborns included blood chemistries (complete blood count and differential, platelet count, liver function tests, troponine I, AAV9-Ab IgG titers); neurological examination of the newborn included standardized assessments of motor function and overall psychomotor development by the Children’s Hospital of Philadelphia Infant.

Test of Neuromuscular Disorder (CHOP-INTEND) scale, Hammersmith Neonatal Neurological Examination (HNNE) and the Bayley III scale of Infant and Toddler Development—third edition.

All patients diagnosed with SMA were treated pharmacologically by day 40 of life (symptomatic patients by day 17 of life). Follow-up was scheduled for all patients.

Based on *SMN2* copy number (1–3 copies), 70% of positive infants would have been eligible for gene therapy; however, due to parental will or high titers of anti-AAV9 antibodies, 30% were treated with nursinersen or risdiplam. Overall, 40% of SMA infants were treated with risdiplam and 10% with nursinersen. Follow-up was scheduled every 2–3 months for Class 2 infants. Follow-up assessments included neurological examination with standardized tests (CHOP intend, Bayley III after 6 months), electroneurography, polysomnography, multidisciplinary assessment (pneumology, neonatology/pediatrics, cardiology). In all cases, physiotherapy, neuropsychomotor therapy and respiratory physiotherapy were undertaken. In no case was respiratory support necessary. All patients showed improvement in motor function. Follow up was schedules every 2–6 months for Class 1 infants, based on the clinical assessment of the individual patient. Follow-up evaluations included neurological examination with standardized tests (CHOP mean, Bayley III after 6 months), electroneurography. In no case were other multidisciplinary evaluations necessary. In only one case was neuropsychomotor therapy undertaken for mild delay in psychomotor acquisitions, recovered within the first year of life.

Since we propose a new evidence-based classification, it can certainly be applied to affected infants reported by other Italian centers that have activated SMA NBS in recent years (34, 35).

Table 1 summarizes clinical/genetic features of our positive SMA newborns, with the new classification and the therapy applied.

**Table 1:** Demographic and clinical characteristics of positive SMA children identified by our NBS program.

Demographic and clinical characteristics	*n* (%)
Females	7 (70%)
Males	3 (30%)
Age at diagnosis (days), mean ± SD	11 ± 1.7
Age at first treatment (days), mean ± SD	17.3 ± 9.1
1–3 copies of SMN2	7 (70%)
≥ 4 copies of SMN2	3 (30%)
Class 1	3 (30%)
Class 2	7 (70%)
Class 3	0 (0%)
Onasemnogene abeparvovec	5 (50%)
Nusinersen	1 (10%)
Risdiplam	4 (40%)

**Figure 4:**
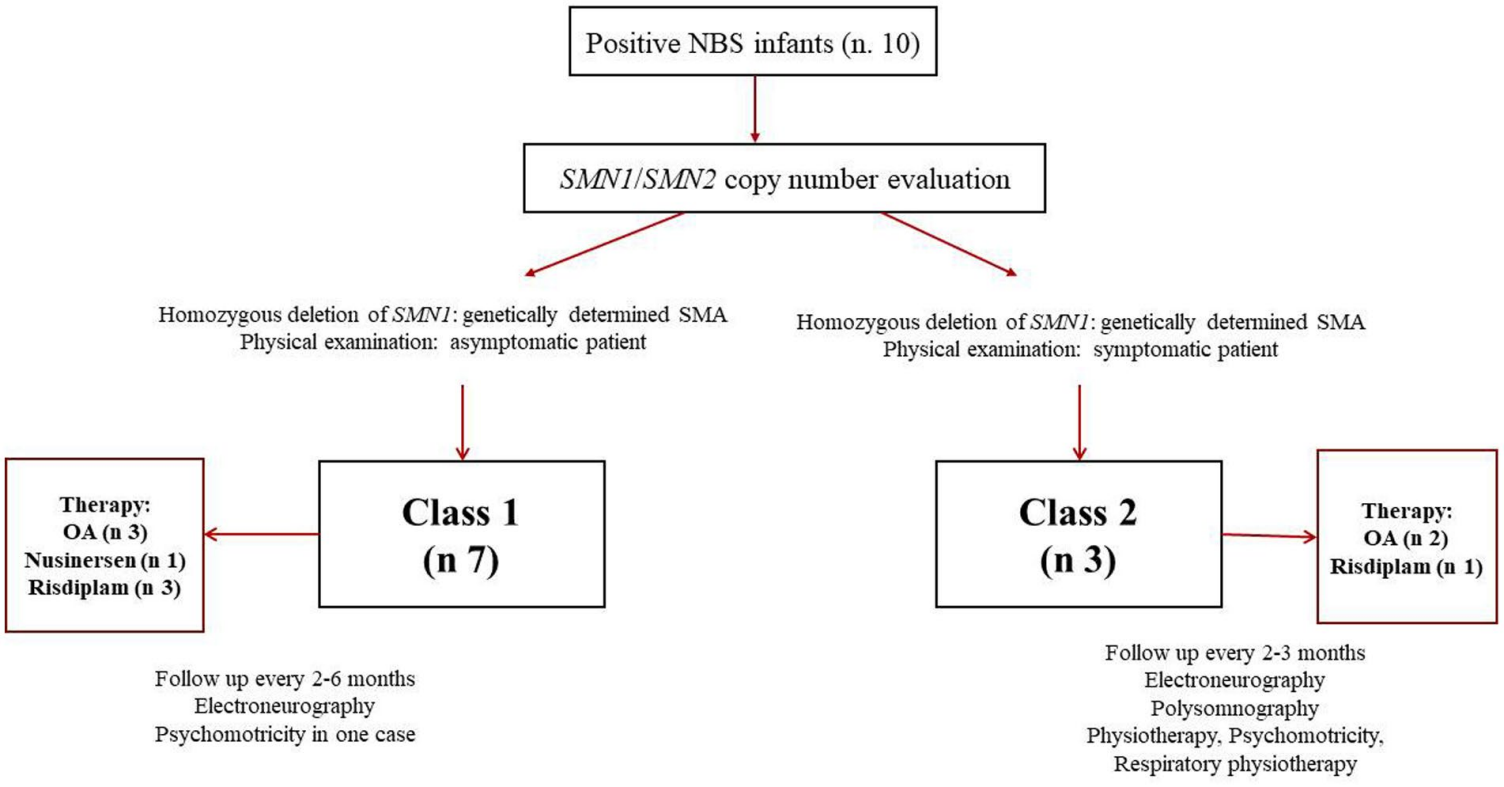
Diagram illustrating application of the proposed new classification to SMA patients identified by newborn screening (NBS). OA: onasemnogene abeparvovec.

Figure 4 reports a diagram illustrating the application of our nomenclature in clinical practice.

## 6 Conclusion

In the last few years, the availability of new and effective therapies and the implementation of NBS with the possibility of early interventions have led to a radical change in the natural history of SMA. However, these improvements also bring uncertainties and challenges. Current challenges include defining appropriate therapeutic and prognostic expectations. We know that *SMN2* copy number, presence of symptoms and disease status, and CMAP are possible prognostic measures. However, how these factors interact with each other to determine an individual clinical course remains unclear. Determining *SMN2* copy number in the diagnostic confirmation process is certainly a primary factor to guide the most suitable therapeutic choice. At the same time, the genotype should be correlated to clinical aspects very quickly. Notably, SMA patients identified by NBS tests should ideally be treated within 14 days of life (36, 37). The choice of therapy is often driven primarily by caregivers’ expectations. In addition to efficacy, the route of administration, frequency of administration, and duration of therapy also impact the decision-making process. To date, there is no reliable data on the long-term efficacy and safety of each of the available therapies. These uncertainties require careful and specific follow-up planning for the individual clinical picture. In the post-NBS era, understanding the variability of clinical course is the basis for setting therapeutic expectations, anticipating comorbidities and ensuring the best care. Therefore, a new classification system in SMA is needed, away from the traditional subdivision based on motor milestones achieved and age of symptom onset. The emergence of new phenotypes requires an update of the nomenclature and a new consensus on multidisciplinary management.

A contemporary classification should include the modality of diagnosis, genotypic characteristics and clinical situation at the time of diagnosis in order to guide therapeutic and prognostic algorithms.

To date, still a low percentage of newborns are screened for SMA in European countries. However, it is increasingly clear that SMA NBS is a necessity, which allows a moderately optimistic view on the extension of such screening to all European countries. In cases where NBS is active, a new classification for SMA infants should already be applied. We hope that our proposal, if considered valid by the main reference centres involved in diagnosis and care of SMA patients, can lay the foundation for the definition of a new and more realistic universal classification, while waiting to know the new “treatment-related” phenotypes of the disease.

In conclusion, we herein propose a new classification aligned with genetic and clinical features of SMA infants identified through NBS, and more objective and specific diagnostic criteria that, if agreed by international consensus, could reduce heterogeneity in clinical practice.
